# UCP1 Dependent and Independent Thermogenesis in Brown and Beige Adipocytes

**DOI:** 10.3389/fendo.2020.00498

**Published:** 2020-07-28

**Authors:** Kenji Ikeda, Tetsuya Yamada

**Affiliations:** Department of Molecular Endocrinology and Metabolism, Tokyo Medical and Dental University, Bunkyo, Japan

**Keywords:** thermogenic fat, brown adipocyte, beige adipocyte, adipogenesis, uncoupling protein 1

## Abstract

Mammals have two types of thermogenic adipocytes: brown adipocytes and beige adipocytes. Thermogenic adipocytes express high levels of uncoupling protein 1 (UCP1) to dissipates energy in the form of heat by uncoupling the mitochondrial proton gradient from mitochondrial respiration. There is much evidence that UCP1 is the center of BAT thermogenesis and systemic energy homeostasis. Recently, UCP1 independent thermogenic pathway identified in thermogenic adipocytes. Importantly, the thermogenic pathways are different in brown and beige adipocytes. Ca^2+^-ATPase 2b calcium cycling mechanism is selective to beige adipocytes. It remains unknown how the multiple thermogenic mechanisms are coordinately regulated. The discovery of UCP1-independent thermogenic mechanisms potential offer new opportunities for improving obesity and type 2 diabetes particularly in groups such as elderly and obese populations who do not possess UCP1 positive adipocytes.

## Thermogenic Fat: Brown and Beige Adipocytes

Mammals have brown and beige thermogenic adipocytes, which are both rich in mitochondria and express uncoupling protein 1 (UCP1). However, brown and beige adipocytes play distinct developmental and anatomical roles in rodents and humans. Brown adipocytes are located in the interscapular and perirenal regions of rodents and infants. By contrast, beige adipocytes (or brite adipocytes) are induced thermogenic adipocytes found sporadically within the white adipose tissue (WAT). The development of beige adipocytes is called “browning” or “beiging.” Beige adipocytes are induced by environmental stimuli, such as chronic cold, β3-adrenergic receptor agonists, peroxisome proliferator-activated receptor gamma (PPARγ) agonists, exercise (1), and cachexia (2). Since the emergence of evidence that adult humans have brown adipose tissue (BAT) (3–7), the debate over whether adult humans have beige adipocytes has been crucial to the metabolic field.

The function of BAT is to regulate the systemic energy balance through non-shivering thermogenesis (NST). Transcriptional analysis of adult human BAT revealed expression of molecular markers specific for murine beige adipocytes (8–10). By contrast, the deep neck region in adult humans possesses thermogenic fat that is similar to the brown fat of mice (11). Of note, clonal analysis of adipose tissue from adult humans revealed evidence that humans have beige adipocytes (12). Even adults who do not exhibit brown fat by ^18^F-labeled fluorodeoxyglucose positron emission tomography/computerized tomography (^18^F-FDG-PET/CT) develop thermogenic fat upon prolonged cold stimulation (13–15).

**Figure 1:**
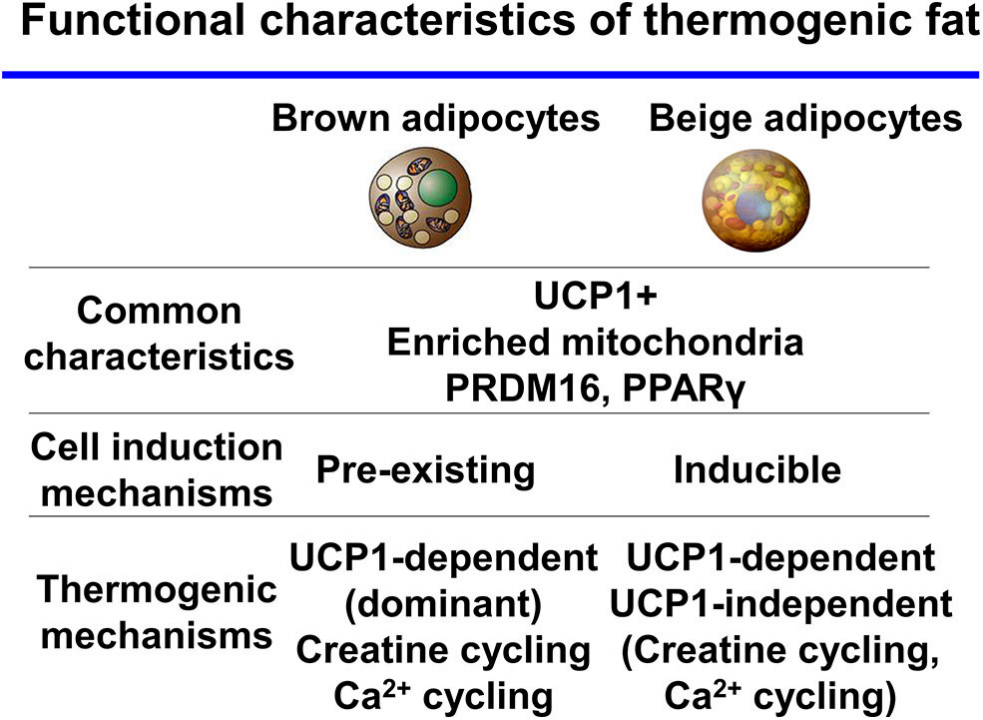
Functional characteristics of thermogenic fat brown and beige adipocytes share many characteristics. In contrast, thermogenic mechanisms are discrete between the two cell types.

Many studies have reported that the amount of cold-induced thermogenic fat positively correlates with the degree of NST and improvements in insulin sensitivity in humans (13–16). Recently, a study showed a much wider distribution of BAT, including in the abdominal subcutaneous regions, in adult humans by refined ^18^F-FDG-PET/CT imaging (17). These results support the existence of thermogenic adipocytes in adult humans. Thermogenic adipocytes have the characteristics of the beige-like inducible adipocytes that contribute to whole-body energy homeostasis. Based on these findings, researchers have hypothesized that beige fat may be a promising new therapeutic target to increase energy expenditure and treat obesity and type 2 diabetes. Future studies will determine the function and distinct, essential characteristics of beige fat in humans. While brown and beige adipocytes share many characteristics such as express UCP1, enriched mitochondria, and differentiation mechanisms-transcriptional factor PR domain-containing protein 16 (PRDM16), PPARγ. In contrast to this, recent studies, mainly in mice, suggest discrete thermogenic mechanisms in the two cell types (Figure 1). In this review, we discuss thermogenic mechanisms and pathways in thermogenic fat.

## UCP1-Dependent Thermogenesis in Thermogenic Fat: Brown and Beige Adipocytes

UCP1 localizes to the mitochondrial inner membrane. It generates heat by dissipating the energy proton gradient from the electron transport chain in mitochondrial respiration. There is considerable evidence that UCP1 is at the center of BAT thermogenesis and systemic energy homeostasis. Many studies have investigated if UCP1 is essential to thermogenesis in thermogenic adipocytes. Ucp1 knockout (KO) mice are unable to maintain their body temperature and develop hypothermia upon acute cold challenge (18). In addition, BAT-deficient mice created by transgenic expression of diphtheria toxin showed diabetic and obese phenotypes in room-temperature environments (19).

The re-synthesis of triacylglycerols after lipolysis is a thermogenic process that is dependent on the amount of ATP needed for triacylglycerol synthesis. Fatty acid synthesis and oxidation are both stimulated and tightly regulated by adrenergic activation (20). Upon adrenergic stimulation, brown adipocyte lipolysis and mitochondrial respiration are activated in a UCP1-dependent manner (21).

However, recent studies in mice with BAT-specific deficiencies in the lipolysis enzyme adipose triglyceride lipase (ATGL) or the ATGL-activating protein comparative gene identification-58 (CGI-58) revealed that the absence of lipolysis in BAT does not change NST (22, 23). These results suggest the existence of compensatory pathways that require further investigation.

## UCP1 Is Dispensable for Thermogenesis in Thermogenic Fat

UCP1, often called thermogenin, had been thought to be the only thermogenic protein responsible for NST in thermogenic fat (24, 25). However, the Kozak group demonstrated that mixed strain F1 *Ucp1* KO mice were able to adapt to cold exposure with gradual acclimation (26–30). Recently, our study revealed that, in an increased beige fat-enriched mouse model, fatty acid-binding protein (aP2)-promoter *Prdm16* transgenic mice (aP2-PRDM16) transgenic × *Ucp1* KO mice could maintain their temperature in a cold environment although mice totally lacking Ucp1 could not (31). This finding suggests the existence of physiologically relevant UCP1-independent thermogenesis in adipocytes.

Intriguingly, *Ucp1* KO mice fed a high-fat diet (HFD) were resistant to the development of obesity at room temperature, suggesting the activation of a UCP1-independent thermogenic pathway (18, 32). Skeletal muscle has mainly been thought to contribute to UCP1-independent thermogenesis via increased capacity for shivering thermogenesis caused by chronic contractile activity (24). However, the findings of the studies on skeletal muscle UCP1-independent thermogenesis are inconsistent and the mechanism requires further investigation (24, 30, 33–36).

## UCP1-Independent Thermogenic Mechanisms in Thermogenic Fat

UCP1 has been thought to be responsible for regulating the energy expenditure and glucose homeostasis of brown and beige fat. The beige fat-deficient adipocyte-specific *Prdm16* KO and adipocyte-specific euchromatic histone-lysine *N*-methyltransferase 1 (EHMT1) KO mice have obese and diabetic phenotypes at room temperature due to insulin resistance (37, 38). However, *Ucp1* KO mice do not have a diabetic phenotype and only develop an obese phenotype under thermoneutral conditions (18, 39). This discrepancy in the metabolic phenotypes of *Ucp1* KO and beige fat-deficient mice suggests that brown and beige fat have UCP1-independent metabolic mechanisms that contribute to systemic energy and glucose homeostasis.

Many observations support the existence of UCP1-independent metabolic mechanisms. The inguinal WAT of *Ucp1* KO mice maintained in a chronic cold environment showed greater respiration than that of *Ucp1* KO mice maintained under thermoneutrality (30). In addition, chronic β3 adrenergic agonist treatment increased oxygen consumption in the epididymal WAT of *Ucp1* KO mice (20). Recently, creatine-substrate cycling (28, 40, 41) and Ca^2+^ cycling have been identified as UCP1-independent thermogenic pathways.

### Creatine-Substrate Cycling

A decline in creatine levels has been linked to the inhibition of thermal responses through unknown mechanisms in rodent models (42, 43). Kazak et al. recently found that creatine substrate cycling stimulates mitochondrial respiration and serves as a thermogenic pathway in thermogenic adipocytes (28, 41). This pathway was discovered in the mitochondria of murine beige fat. Recently, the creatine thermogenic pathway has been suggested to exist in other adipocytes because fat-specific deletion of the creatine synthesis rate-limiting enzyme glycine amidinotransferase (Gatm) reduced creatine levels in BAT and conferred mild cold intolerance (40). Global creatine transporter (*Slc6a8*) KO mice had similar levels of creatine reduction as the adipocyte-specific *Gatm* KO mice, and had an obese phenotype compared to controls (44). Similarly, creatine enzyme *Ckmt1* and *Ckb* double KO mice showed cold intolerance and reduced norepinephrine responses to activate thermogenic respiration (45). These data support the role of the creatine substrate cycling pathway as an adaptive thermogenesis pathway *in vivo*.

### UCP1-Independent Thermogenesis: Ca^2+^-Dependent ATP Hydrolysis in Brown Adipocytes and Muscle

Calcium transport contributes to NST in both BAT and muscle through sarco-endoplasmic reticulum ATPase (SERCA) activity (46–48). In muscle, Ca^2+^ cycling pathways involving SERCA drive thermogenesis such as malignant hyperthermia. Ca^2+^ cycling in the extraocular heater muscle cells of fish suggests the process may be evolutionarily conserved (49, 50).

Sarcolipin (SLN) is a direct peptide-binding SERCA that localizes to the sarcoplasmic reticulum of skeletal muscle. SLN regulates SERCA-mediated ATP turnover in muscle via Ca^2+^ cycling without affecting ATPase activity (51). SLN may function as an uncoupler of calcium transport from ATP hydrolysis via SERCA, which would be elevated in NST (52). The physiological role of SLN in NST is supported by several mouse studies. *Sln* KO animals are mildly cold-intolerant (53). In a mouse model of surgical intrascapular BAT ablation, the removal of BAT was tolerated in the setting of acute cold exposure. By contrast, intrascapular BAT ablation in *Sln* KO mice resulted in cold intolerance, despite the maintenance of skeletal muscle shivering (53). Moreover, *Sln* KO mice fed a HFD had an obese phenotype, whereas mice with muscle-specific transgenic expression of *Sln* fed a HFD had an obesity-resistant phenotype (54, 55). These data support the role of SLN in regulating systemic energy expenditure via calcium uncoupling (53).

### UCP1-Independent Calcium Cycling Thermogenic Mechanisms in Beige Adipocytes

**Figure 2:**
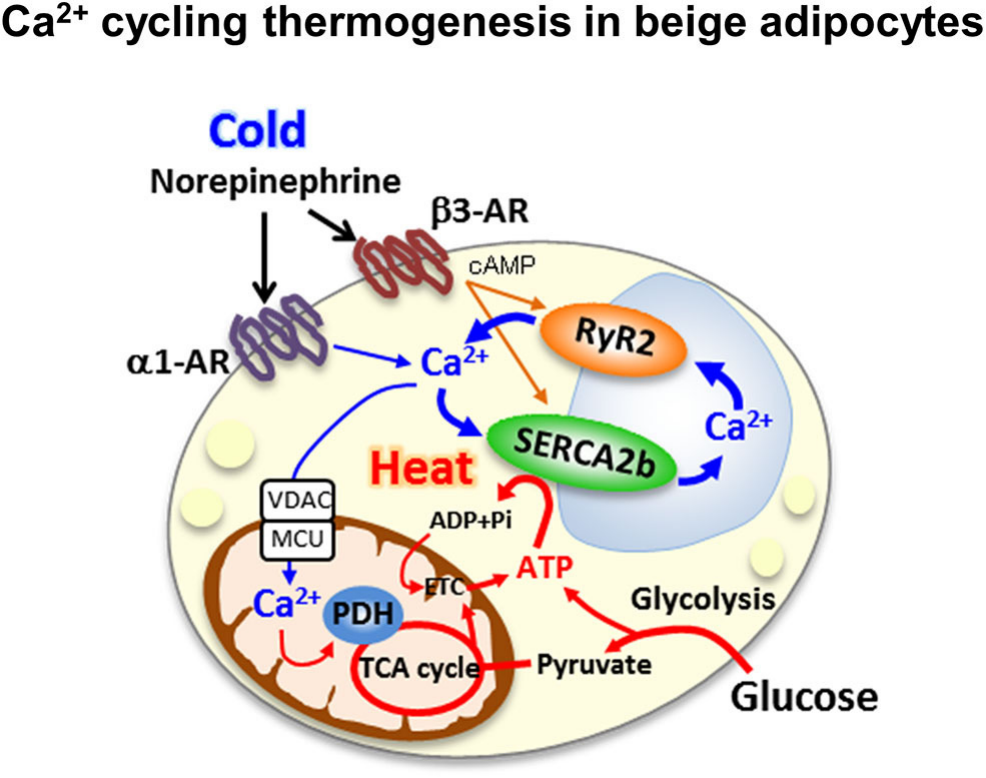
Ca^2+^ cycling thermogenesis in beige adipocytes the newly identified UCP1-independent thermogenic mechanism depends on ATP-dependent Ca^2+^ cycling via sacro-endoplasmic reticulumn ATPase 2b(SERCA2b) and the Ca^2+^ release channel ryanodine receptor 2 (RyR2).

Our recent study revealed a new thermogenic mechanism in beige adipocytes. The newly identified UCP1-independent thermogenic mechanism depends on ATP-dependent Ca^2+^ cycling via SERCA2b and the Ca^2+^ release channel ryanodine receptor 2 (RyR2) (Figure 2) (31). Adipocyte-specific *Serca2* KO mice have impaired beige adipocyte thermogenesis. Intriguingly, the SERCA2b-mediated Ca^2+^ cycling thermogenic mechanism is necessary for beige adipocyte thermogenesis, but dispensable in brown adipocytes. The selectivity of this pathway for beige fat relate to the ability of beige adipocytes to produce ATP due to their high expression of ATP synthase. Thus, beige adipocytes can generate heat in an ATP-dependent manner through SERCA2b-mediated Ca^2+^ cycling even in the absence of UCP1. Brown adipocytes express low levels of ATP synthase and cannot produce ATP due to their low ATP synthesis capacity (56).

Furthermore, our study showed that beige fat dramatically contributes to whole-body energy and glucose homeostasis via UCP1-independent metabolic mechanisms. Mice with beige fat-specific overexpression of Prdm16 driven by the aP2 promoter were protected from diet-induced obesity compared to littermate control mice (57). Furthermore, aP2-*Prdm16* transgenic × *Ucp1* KO mice fed a HFD were resistant to obesity, even in the absence of UCP1 (31). Importantly, both aP2-*Prdm16* transgenic mice and aP2-*Prdm16* transgenic × *Ucp1* KO mice showed dramatically better glucose homeostasis on a HFD than mice with normal *Prdm16* expression. These data strongly support the existence of a UCP1-independent metabolic mechanism in beige fat that contributes to systemic energy and glucose homeostasis. Therefore, Ca^2+^ cycling mediated by SERCA2–RyR2 signaling in beige adipocytes may be a potential therapeutic target for obesity and type 2 diabetes. For example, S107, a pharmacological RyR2 stabilizer that minimizes Ca^2+^ leak from RyR2 and increases Ca^2+^ loading from the endoplasmic reticulum (58), enhances Ca^2+^ cycling thermogenesis. S107 treatment of *Ucp1* KO mice induced resistance to hypothermia upon cold exposure by activating UCP1-independent thermogenesis (31).

Nevertheless, there is concern that activation of Ca^2+^ cycling *in vivo* may have potentially harmful effects on skeletal muscle and the heart. *Ryr1* mutation causes malignant hyperthermia (50), and human *RYR2* gene mutations cause arrhythmogenic right ventricular cardiomyopathy type 2 and lethal arrhythmia due to catecholaminergic polymorphic ventricular tachycardia (59, 60). Given that activating systemic Ca^2+^ cycling may be harmful, it may be promising to activate Ca^2+^ cycling selectively in beige fat to treat obesity and type 2 diabetes while avoiding harmful effects on the muscle and heart.

## Beyond Thermogenesis in Thermogenic Adipocytes

Recently, some studies shed light on the physiological function of beige fat to repress adipose tissue fibrosis; these findings are likely to extend beyond thermogenesis (61). Chronic cold-acclimated mice or mice with adipocyte-specific *Prdm16* overexpression markedly repress adipose tissue fibrosis. Of note, this repressive effect was independent of UCP1 and independent of body weight reduction (62). The repression of adipose tissue fibrosis caused notable improvements in systemic glucose homeostasis via a UCP1-independent mechanism (62). Although the findings need to be supported by further work, it appears that beige fat can repress adipose tissue fibrosis and control whole-body glucose homeostasis. Brown and beige fat release several physiological agents, known as “batokines,” to control systemic glucose homeostasis (63–66). These data suggest that thermogenic fat has an important physiological function beyond thermogenesis.

## Discussion

As thermogenic adipocytes exert multiple thermogenic mechanisms, it will be critical to determine how the regulation of the multiple mechanisms is orchestrated. SLN, a crucial calcium uncoupler, is not expressed in beige adipocytes (31); beige adipocytes must utilize an unknown regulator of the calcium system. Further work is needed to determine the regulator of SERCA2B activity and calcium uncoupler in beige adipocytes.

Brown adipocytes and beige adipocytes have common characteristics, but recent evidence indicates that they have distinct thermogenic mechanisms and functions. Of note, UCP1 is still the main regulator of thermogenesis in BAT, as revealed by numerous studies. However, emerging evidence suggests that beige fat uses UCP1-independent thermogenic pathways, which substantially contribute to systemic energy homeostasis. It will be essential to determine the coordination and contribution of the canonical (UCP1-dependent) and non-canonical (UCP1-independent) thermogenic mechanisms in adipose tissue to whole-body energy homeostasis. In particular, the newly identified UCP1-independent thermogenic pathways creatine substrate cycling and Ca^2+^ cycling should be evaluated as non-canonical mechanisms of thermogenesis.

Intriguingly, Ca^2+^ cycling-related thermogenesis seems to be evolutionarily conserved in humans and mice, and even in species that lack functional UCP1, such as pigs (31). These data suggest that UCP1-independent Ca^2+^ cycling thermogenesis may be the fundamental thermogenic system. Importantly, fibroblast growth factor 21 (FGF21) signaling increases intracellular Ca^2+^ levels in adipocytes (67) and induces browning (68). Recent evidence indicates that the anti-obesity and anti-diabetic activities of FGF21 are UCP1-independent (69, 70). Furthermore, FGF21 and UCP1 are not required for cold environment acclimation in mice (71). These findings suggest that at least some of the metabolic actions of FGF21 are mediated via UCP1-independent thermogenesis in adipose tissue.

It is of high clinical importance to determine the regulator of UCP1-independent thermogenesis because understanding the mechanism may lead to the development of new treatments for obesity and type 2 diabetes. This may be promising for treating obese and elderly populations who do not possess UCP1-positive adipocytes. The current literature suggests that it may be possible to selectively activate UCP1-independent thermogenesis, such as that mediated by Ca^2+^ cycling, to treat patients who lack UCP1-positive adipocytes. New tools such as “designer receptors exclusively activated by designer drugs” (DREADD) and the optogenetic tool channel rhodopsin 2 (ChR2) (72) may modulate intracellular calcium signaling pathways in adipocytes and lead to novel treatments for obesity and type 2 diabetes.

## Author Contributions

KI and TY wrote the manuscript and edited the manuscript. All authors contributed to the article and approved the submitted version.

## Conflict of Interest

The authors declare that the research was conducted in the absence of any commercial or financial relationships that could be construed as a potential conflict of interest.
